# Commentary: Diagnosing Diabetic Retinopathy With Artificial Intelligence: What Information Should Be Included to Ensure Ethical Informed Consent?

**DOI:** 10.3389/fmed.2021.765936

**Published:** 2021-11-24

**Authors:** Michael D. Abramoff, Zachary Mortensen, Chris Tava

**Affiliations:** ^1^Deartments of Ophthalmology and Visual Sciences, University of Iowa, Iowa City, IA, United States; ^2^Digital Diagnostics Inc., Coralville, Iowa City, IA, United States

**Keywords:** healthcare, autonomous, artificial intelligence, informed consent, patient outcome, equity, bias

## Introduction

The recent paper by Ursin et al. (1) brings up crucial issues about the ethics of healthcare AI and more specifically autonomous AI. These issues include the responsibilities and liabilities of disclosing information to patients. We appreciate the authors illustrating these issues with IDx-DR, which as the first autonomous AI approved by US Food and Drugs Administration (FDA), crystallized so many issues around “a computer making a medical decision,” as the authors carefully point out.

## Ethics in Healthcare Ai

During the development, validation, and implementation of IDx-DR (Digital Diagnostics Inc), we started with an ethical framework built on the principles of non-maleficence, autonomy, and justice, which continue to be developed in various publications (2–4). This framework made it possible to track metrics around safety, equity, efficiency, transparency, validability, and accountability, allowing AI to be done the right way. This has led to validation of this biomarker-based AI under FDA oversight, inclusion in standards of care. An important milestone was reimbursement at the $55 level by publicly funded insurance in the United States. This required an understanding of the value of “autonomous AI *work*” by all stakeholders, and has led to rapid and increasingly widespread implementation (5–7). The ethical framework thus continues to serve all stakeholders well, as we continue to jointly develop considerations and requirements for healthcare AI.

In this context, it is interesting to contrast healthcare autonomous AI, to another type of digital technology, social media. Healthcare autonomous AI was grounded from the start in an ethical framework, and the technology stack was then built according to this framework. Social media, instead, started with the tech, and only now, almost two decades later, are we starting to grapple with its ethical consequences.

## Clarifying Several Issues of Note

a. **IDx-DR is a fully autonomous AI system**. While the authors refer to IDx-DR as “AI-aided DR diagnosis,” it is in fact a fully autonomous AI system, as explained above. As a consequence, Digital Diagnostics assumes liability for the performance of the AI, as is now also required by the American Medical Association's AI Policy (8). We remain convinced that clarifying this liability issue helps foster acceptance by physicians and other stakeholders.
b. **AI bias**. The authors rightfully bring up the problem of undesirable bias, including racial and ethnic bias. In Digital Diagnostics' ethical framework, including metrics for equity, we recognize that the bias problem applies to the entire AI lifecycle. This includes choice of disease and disease severity to be diagnosed, AI algorithm design, including the use of priors such as biomarkers, instead of prior-less blank slate black box algorithms, the distribution of the training sets, rigorous validation for improved outcome metrics including equity, and the choice of where it is implemented after regulatory approval (2, 9). As illustrated in these studies, IDx-DR is a biomarker based AI system, and explicitly not a black box system, avoiding the latter's' many risks, including catastrophic failure and risk of bias (10–12).
c. **Patient informed consent**. The authors are correct that informed consent of patients, notifying them that an AI will be used, should be considered. For IDx-DR, both operators of the AI system, as well as the physicians ordering it, are trained in how to discuss the use of IDx-DR with patients. In fact, Digital Diagnostics has developed an AI facts label as part of the diagnostic output, so as to maximize transparency about which AI algorithms are used, their accuracy, and the relevant scientific evidence of their use and benefit.
d. **CE Certification**. Finally, IDx-DR was for autonomous use in the European Economic Area per its CE Certificate (13) and complies with GDPR Article 22.

## Author Contributions

Ethics in healthcare AI section attributed to MDA. Section A: IDx-DR is a fully autonomous AI system was written by CT and ZM. Section B: AI bias was written by MDA and CT. Section C: Patient informed consent was written by MDA and ZM. Section D: CE certification was written by MDA. All authors contributed to the article and approved the submitted version.

## Funding

This work was supported in part by the Robert C. Watzke MD Professorship (to MDA) and Research to Prevent Blindness, Inc., New York, New York (unrestricted grant to the Department of Ophthalmology, and Visual Sciences, University of Iowa.

## Conflict of Interest

MDA is a founder, executive chairman, consultant, investor, and shareholder of Digital Diagnostics. CT is a shareholder of Digital Diagnostics. The remaining author declares that the research was conducted in the absence of any commercial or financial relationships that could be construed as a potential conflict of interest.

## Publisher's Note

All claims expressed in this article are solely those of the authors and do not necessarily represent those of their affiliated organizations, or those of the publisher, the editors and the reviewers. Any product that may be evaluated in this article, or claim that may be made by its manufacturer, is not guaranteed or endorsed by the publisher.

## References

[1] Ursin F Timmermann C Orzechowski M Steger F. Diagnosing diabetic retinopathy with artificial intelligence: what information should be included to ensure ethical informed consent? Front Med. (2021) 8:1108. 10.3389/fmed.2021.695217 34368192 PMC8333706

[2] Abramoff MD Tobey D Char DS. Lessons learned about autonomous AI: finding a safe, efficacious, and ethical path through the development process. Am J Ophthalmol. (2020) 214:134–42. 10.1016/j.ajo.2020.02.022 32171769

[3] Char DS Abràmoff MD Feudtner C. Identifying ethical considerations for machine learning healthcare applications. Am J Bioethics. (2020) (2020) 20:7–17. 10.1080/15265161.2020.1819469 33103967 PMC7737650

[4] Abramoff MD Cunningham B Patel B Eydelman MB Leng T Sakamoto T. Foundational Considerations for Artificial Intelligence. Ophthalmology. (2021). 10.1016/j.ophtha.2021.08.023. [Epub ahead of print]. 34478784 PMC9175066

[5] American Diabetes A. 11. Microvascular complications and foot care: standards of medical care in diabetes-2020. Diabetes Care. (2020) 43(Suppl. 1):S135–51. 10.2337/dc20-S011 31862754

[6] U.S. Food & Drug Administration (FDA). FDA Permits Marketing of Artificial Intelligence-Based Device to Detect Certain Diabetes-Related Eye Problems. (2018). Available online at: https://www.fda.gov/newsevents/newsroom/pressannouncements/ucm604357.htm

[7] Centers for Medicare & Medicaid Services. Proposal to Establish Values for Remote Retinal Imaging (CPT Code 92229). (2021). 56ff. Available online at: https://public-inspection.federalregister.gov/2021-14973.pdf

[8] American Medical Association (AMA) Board of Trustees Policy Summary. Augmented Intelligence in Healthcare. (2019). Available online at: https://www.ama-assn.org/system/files/2019-08/ai-2018-board-policy-summary.pdf

[9] Abràmoff MD Lavin PT Birch M Shah N Folk JC. Pivotal trial of an autonomous AI-based diagnostic system for detection of diabetic retinopathy in primary care offices. Nat Digital Med. (2018) 1:39. 10.1038/s41746-018-0040-6 31304320 PMC6550188

[10] Finlayson SG Bowers JD Ito J Zittrain JL Beam AL Kohane IS. Adversarial attacks on medical machine learning. Science. (2019) 363:1287–9. 10.1126/science.aaw4399 30898923 PMC7657648

[11] Shah A Lynch S Niemeijer M Amelon R Clarida W Folk J. Susceptibility to misdiagnosis of adversarial images by deep learning based retinal image analysis algorithms. In: Proceedings – International Symposium on Biomedical Imaging (2018).

[12] Lynch S Abramoff MD. Catastrophic failure in image-based convolutional neural network algorithms for detecting diabetic retinopathy. IOVS. (2017) 58:3776.

[13] UL CE Mark Certification. Certificate of Registration. (2018). Available online at: https://database.ul.com/certs/PDWS.A18142.pdf

